# Neuroprotective effect of bilberry extract in a murine model of photo-stressed retina

**DOI:** 10.1371/journal.pone.0178627

**Published:** 2017-06-01

**Authors:** Hideto Osada, Tomohiro Okamoto, Hirohiko Kawashima, Eriko Toda, Seiji Miyake, Norihiro Nagai, Saori Kobayashi, Kazuo Tsubota, Yoko Ozawa

**Affiliations:** 1 Laboratory of Retinal Cell Biology, Department of Ophthalmology, Keio University School of Medicine, Tokyo, Japan; 2 Department of Ophthalmology, Keio University School of Medicine, Tokyo, Japan; 3 Wakasa Seikatsu Co., Ltd., Kyoto, Japan; University of Florida, UNITED STATES

## Abstract

Excessive exposure to light promotes degenerative and blinding retinal diseases such as age-related macular degeneration and retinitis pigmentosa. However, the underlying mechanisms of photo-induced retinal degeneration are not fully understood, and a generalizable preventive intervention has not been proposed. Bilberry extract is an antioxidant-rich supplement that ameliorates ocular symptoms. However, its effects on photo-stressed retinas have not been clarified. In this study, we examined the neuroprotective effects of bilberry extract against photo-stress in murine retinas. Light-induced visual function impairment recorded by scotopic and phototopic electroretinograms showing respective rod and cone photoreceptor function was attenuated by oral administration of bilberry extract through a stomach tube in Balb/c mice (750 mg/kg body weight). Bilberry extract also suppressed photo-induced apoptosis in the photoreceptor cell layer and shortening of the outer segments of rod and cone photoreceptors. Levels of photo-induced reactive oxygen species (ROS), oxidative and endoplasmic reticulum (ER) stress markers, as measured by real-time reverse transcriptase polymerase chain reaction, were reduced by bilberry extract treatment. Reduction of ROS by N-acetyl-L-cysteine, a well-known antioxidant also suppressed ER stress. Immunohistochemical analysis of activating transcription factor 4 expression showed the presence of ER stress in the retina, and at least in part, in Müller glial cells. The photo-induced disruption of tight junctions in the retinal pigment epithelium was also attenuated by bilberry extract, repressing an oxidative stress marker, although ER stress markers were not repressed. Our results suggest that bilberry extract attenuates photo-induced apoptosis and visual dysfunction most likely, and at least in part, through ROS reduction, and subsequent ER stress attenuation in the retina. This study can help understand the mechanisms of photo-stress and contribute to developing a new, potentially useful therapeutic approach using bilberry extract for preventing retinal photo-damage.

## Introduction

Photons are indispensable for visual function; however, excessive exposure may overwhelm the receptor capacity of the retina, resulting in stress and degeneration of the tissue. Retinal damage can be caused by staring at the sun, termed solar retinopathy, as well as staring at computer monitors while playing games [1]. Retinitis pigmentosa [2] and age-related macular degeneration [3, 4] are the leading causes of blindness and are characterized by retinal degeneration exacerbated by light exposure [5–11]. Thus, preventive interventions for reducing photo-stress have been suggested in order to lower the risk of disease progression. However, no specific treatment has been established except for blocking light using glasses, which may not be helpful for patients with sub-average visual function who may require bright light to recognize targets.

Previous reports have suggested that rhodopsin metabolism plays a role in photo-induced retinal degeneration [12, 13]. Although reactive oxygen species (ROS) are generated during normal physiological metabolism, intrinsic mechanisms are involved in the maintenance of homeostasis. However, when ROS levels exceed processing capacity, they cause abnormal modifications of cellular components, such as proteins, and subsequently dysregulate multicellular organelles and induce cellular death [14]. Similarly, excessive light exposure increases ROS generation and causes retinal photoreceptor death [15, 16].

When protein abnormalities are detected in the endoplasmic reticulum (ER), cells maintain homeostasis by triggering an unfolded protein response (UPR) [17, 18], which suppresses overall protein production to prevent the accumulation of abnormally modified proteins. However, when abnormalities exceed the homeostatic capacity of the UPR, cell death signaling molecules such as the CCAAT-enhancer-binding protein (C/EBP) homologous protein (CHOP) are induced to promote the transcription of pro-apoptotic molecules. Therefore, ER stress can be involved in cell death, and the pathogenesis of blindness due to retinitis pigmentosa, an inherited retinal degeneration, at least partly involves ER stress [19–21]. Light exposure-induced ER stress was previously reported in a photoreceptor cell line [22, 23] and in the retinal pigment epithelium (RPE), which maintains photoreceptors, of autophagy-deficient mice [24, 25], and in the neural retina [23]. However, the relationship between ROS accumulation and ER stress in light-induced photoreceptor degeneration is not fully understood.

Bilberry extract is an antioxidant-rich supplement that contains various anthocyanins. It is not only believed to ameliorate ocular symptoms but has also been used in clinical trials for eye fatigue [26]. It can reduce ROS, as shown in *in vitro* experiments [27, 28], and suppress the pathogenesis of innate retinal inflammation [29] and diabetes [30–32] in animal models. However, its effects on photo-stressed retinas have not been clarified. In the present study, we evaluated the preventive effects of bilberry extract on photo-induced retinal degeneration in Balb/c mice. We focused particularly on the degeneration of rod photoreceptor cells, which form the visual field, and cone photoreceptor cells, which determine visual acuity in humans, with an emphasis on local ROS accumulation and ER stress. The influence of ROS on ER stress was further analyzed in the retina using a well-known antioxidant, N-acetyl-L-cysteine (NAC), which reduces retinal ROS [16].

## Materials and methods

### Animals

Seven- to eight-week-old male Balb/c mice (CLEA Japan, Tokyo, Japan) were housed in an air-conditioned room maintained at 22 ± 2°C under a 12-h dark/light cycle, with free access to a standard diet (CLEA Japan, Tokyo, Japan) and tap water. The mice were randomly divided into three groups (non-light exposed control and light-exposed mice treated with vehicle, as well as light-exposed mice treated with either bilberry extract or NAC, as described below). All animal experiments were conducted in accordance with the Association for Research in Vision and Ophthalmology Statement for the Use of Animals in Ophthalmic and Vision Research, and the guidelines of the Animal Care Committee of Keio University. The animal experimental protocols were approved by the Animal Care Committee of Keio University (Approval No. 08002).

### Light exposure

Mice were exposed to light as previously described [15, 33–35]. Briefly, the mice were dark-adapted by maintaining them in complete darkness for 12 h before light exposure. Their pupils were then dilated with a mixture of 0.5% each of tropicamide and phenylephrine (Mydrin-P^®^, Santen Pharmaceutical, Osaka, Japan) just before exposure to light. The mice were exposed to a white fluorescent lamp (FHD100ECW, Panasonic, Osaka, Japan) at 3000 lux for 1 h (starting at 0900) in a dedicated exposure box maintained at 22 ± 2°C containing stainless steel mirrors on each wall and the floor (Tinker N, Kyoto, Japan). After light exposure, the mice were returned to their cages and maintained under a dim cyclic light (5 lux, 12 h on/off) until they were euthanized with sodium pentobarbital (70 mg/kg BW) at specific time points according to the experimental protocol. The control mice without light exposure were also maintained under dim cyclic lighting and were euthanized with sodium pentobarbital (70 mg/kg BW) at corresponding times.

### Administration of bilberry extract and NAC

Bilberry extract (containing about 39% anthocyanins) was provided by Wakasa Seikatsu (Kyoto, Japan), and was dissolved in phosphate-buffered saline (PBS) and orally administered through a stomach tube (750 mg/kg body weight, BW) at 12 h and 30 min before light exposure. Mice receiving vehicle received PBS alone. Alternatively, intraperitoneal injection of NAC (Nakalai Tesque, Kyoto, Japan, 250 mg/kg BW) diluted in PBS was administered at 12 h and 30 min before light exposure. Again, mice receiving vehicle received PBS alone. The dose and time points of bilberry extract and NAC administration were chosen based on optimization studies performed previously by our group [16, 29]. The control mice without light exposure received either two oral administrations or intraperitoneal injections of vehicle at the corresponding time points in the respective experiments.

### Electroretinograms (ERG)

The mice were dark-adapted for at least 12 h and then placed under dim red illumination before conducting ERGs. The mice were anesthetized with 60 mg/kg BW sodium pentobarbital (Dainippon Sumitomo Pharmaceutical Co., Osaka, Japan) and kept on a heating pad throughout the experiment. Mouse pupils were dilated using a single drop of a mixture of 0.5% each of tropicamide and phenylephrine. The ground and reference electrodes were then placed on the tail and in the mouth, respectively, while the active gold wire electrodes were placed on the cornea.

The recordings were made with a PowerLab System 2/25 (AD Instruments, New South Wales, Australia). Full-field scotopic ERGs were measured in response to a flash at intensities ranging of −2.12 to 2.89 log cd s/m^2^. Photopic ERGs were measured after 10 min of light adaptation. Flash stimuli ranging from 0.48 and 1.48 log cd s/m^2^ were used for recording with a background of 30 cd s/m^2^ (PowerLab System 2/25; AD Instruments; New South Wales, Australia), and the results of 20 single-flash trace trials were averaged. The responses were differentially amplified and filtered through a digital bandpass filter ranging from 0.3 to 1000 Hz. Each stimulus was delivered using a commercial stimulator (Ganzfeld System SG-2002; LKC Technologies, Inc., Gaithersburg, MD) and the a-wave amplitude was measured from the baseline to the trough while the b-wave amplitude was measured from the trough of the a-wave to the peak of the b-wave. The implicit times of the a- and b-waves were measured from the onset of the stimulus to the peak of each wave. The peak points were automatically indicated by the system and confirmed by the examiner. The experiments were performed 4 days after light exposure when the photo-stress-induced ERG change was obvious according to our previous reports [15, 33–35].

### Histological analyses

Mouse eyes were enucleated and fixed in 4% paraformaldehyde (PFA) overnight at 4°C. After fixation, the eyes were embedded in paraffin (Sakura Finetek Japan, Tokyo, Japan), and the sections (6- to 8-μm thick), which included the optic nerve head to the most peripheral region of the retina, were deparaffinized by passing them through the following treatment series thrice for 5 min each: xylene, 1:1 xylene-alcohol, 100% ethanol, 90% ethanol, 70% ethanol, and 50% ethanol. The ethanol was then removed by rinsing with distilled water.

Terminal deoxynucleotidyl transferase (TdT)-mediated deoxyuridine triphosphate (dUTP) nick-end labeling (TUNEL) assay was performed using the ApopTag red apoptosis detection kit (Millipore, Bedford, MA, USA) according to the manufacturer’s protocol. The nuclei were stained with Cellstain-4',6-diamidino-2-phenylindole (DAPI) solution (2 μg/mL, Dojindo Molecular Technologies, Kumamoto, Japan). The TUNEL-positive cells in each section were counted and averaged. All the groups were analyzed 2 days after light exposure, which was a time point where a clear change in photoreceptor apoptosis was previously detected [15, 33–35].

To measure the thickness and length of retinal components, sections were stained with hematoxylin and eosin or incubated with either a rabbit anti-rhodopsin antibody (1:10,000; Cosmo Bio Co., Ltd., Tokyo, Japan) or a rabbit anti-blue opsin antibody (1:500; Merck Millipore, Darmstadt, Germany) overnight at 4°C. Antibody-incubated sections were then incubated with Alexa 488-conjugated goat anti-rabbit IgG (Invitrogen Japan, Tokyo, Japan) with subsequent counterstaining with DAPI solution (2 μg/mL) for 1 h at room temperature. Measurements were performed using ImageJ software (developed by Wayne Rasband, National Institutes of Health, Bethesda, MD, USA; available at http://rsb.info.nih.gov/ij/index.html), and averaged as described previously [16, 34, 36, 37]. All the groups were analyzed 4 days after light exposure, which was a time point where clear changes in the photoreceptors were previously detected [15, 33–35].

For double immunostaining of activating transcription factor 4 (ATF4) and glutamine synthetase, sections obtained 12 h after light exposure were immersed in preheated antigen-retrieval Immunosaver solution (Wako, Tokyo, Japan) and boiled for 45 min at 90°C. Next, sections were stained sequentially with a rabbit anti-ATF4 antibody (1:100; CST JAPAN, Tokyo, Japan) and a mouse anti-glutamine synthetase antibody (1:500; BD Transduction Laboratories, San Jose, CA, USA) overnight at 4°C. Signals were obtained using Alexa 488-conjugated goat anti-rabbit IgG and Alexa 555-conjugated goat anti-mouse IgG, respectively, with subsequent counterstaining with DAPI solution (2 μg/mL), all for 1 h at room temperature.

Sections were examined under a microscope equipped with a digital camera (Olympus Co., Tokyo, Japan), and fluorescent images were obtained using a confocal microscope (TCS-SP5; Leica, Tokyo, Japan).

### ROS measurement

The mouse eyes were enucleated and the retinas were immediately frozen and homogenized in PBS using a Mixer Mill MM 300 homogenizer (Qiagen Inc., Chatsworth, CA, USA). The tissue homogenates were incubated with 2’,7’-dichlorofluorescein-diacetate (DCFH-DA) (50 μM, Sigma-Aldrich, St. Louis, MO, USA) at 37°C in the dark for 1 h. Then, the samples were centrifuged at 3000 rpm for 5 min at 4°C. Finally, pellets were washed with cold PBS twice and resuspended in cold PBS. DCFH-DA fluorescence was measured using Synergy4 (BioTek Instruments, Inc., Winooski, VT, USA) at 1, 3, and 6 h after light exposure. The data at 6 h, when significant differences between the groups were observed, are presented.

### Real-time reverse-transcription polymerase chain reaction (RT-PCR)

Total RNA was isolated from mouse retinas or RPE-choroid complex with TRIzol reagent (Life Technologies, Carlsbad, CA, USA). RNA concentration was measured using a NanoDrop 1000 (Thermo Fisher Scientific, Waltham, MA, USA) and 1 μg RNA was reverse-transcribed using the SuperScript VILO master mix (Life Technologies, Carlsbad, CA, USA) according to the manufacturer’s instructions. The following primer sequences were used: glyceraldehyde 3-phosphate dehydrogenase (GAPDH), forward 5'-AACTTCGGCCCCATCTTCA-3' and reverse 5'-GATGACCCTTTTGGCTCCAC-3'; ho-1, forward 5'-ACGCATATACCCGCTACCTG-3' and reverse 5'-CCAGAGTGTTCATTCGAGCA-3'; bip, forward 5'-TGCAGCAGGACATCAAGTTC-3' and reverse 5'-TTTCTTCTGGGGCAAATGTC-3', chop, forward 5'-CTGGAAGCCTGGTATGAGGA-3' and reverse 5'-GGACGCAGGGTCAAGAGTAG-3'; atf4, forward 5'-GAAACCTCATGGGTTCTCCA-3' and reverse 5'-TCCATTTTCTCCAACATCCA-3'; xbp1s, forward 5'-CTGAGTCCGCAGCAGGTG-3' and reverse 5'-TGCCCAAAAGGATATCAGACT-3'; and sec24d, forward 5'-CACCACAGCTCCAGAGGAAG-3' and reverse 5'-GCCCCTGGTATTGGTCGTAT-3'. Real-time PCR was performed using the StepOnePlus^™^ PCR system (Applied Biosystems, Foster City, CA, USA) and gene expression was quantified using the ΔΔCT method. All mRNA levels were normalized to those of GAPDH as described previously [16, 34, 38, 39]. The mRNA levels were measured at 6, 12, and 24 h after light exposure, and the data at 12 h, when significant differences between the groups were observed, are presented.

### Flat mount immunohistochemistry

Mouse eyes were enucleated and the cornea, lens, vitreous, and retina were removed prior to preparing flat-mount eyecups 24 h after light exposure. The eyecups were prefixed with 4% PFA for 1 h at 4°C and flattened by making four radial cuts before being returned to 4% PFA. The samples were blocked with 5% BSA in PBS for 30 min at room temperature and then incubated overnight with anti-ZO-1 antibody (1:500; Invitrogen) at 4°C. Signals were obtained using an Alexa Fluor 488 goat anti-rabbit IgG antibody and DAPI solution (2 μg/mL). Fluorescent images of the flat mounts were obtained using a confocal microscope. The number of intact RPE cells visible under ZO-1 immunostaining of the intracellular face of an entire cell membrane and the total RPE cells were counted in four quadrants of a 400-μm square in the central part of the retina (superior, inferior, nasal, and temporal).

### Statistical analyses

All results are expressed as mean ± standard deviation (SD). A one-way analysis of variance (ANOVA) with the Tukey’s post hoc test was used to assess the statistical significance of the differences, and results with P-values < 0.05 were considered significant.

## Results

### Suppression of photo-induced visual function impairment by bilberry extract

**Fig 1:**
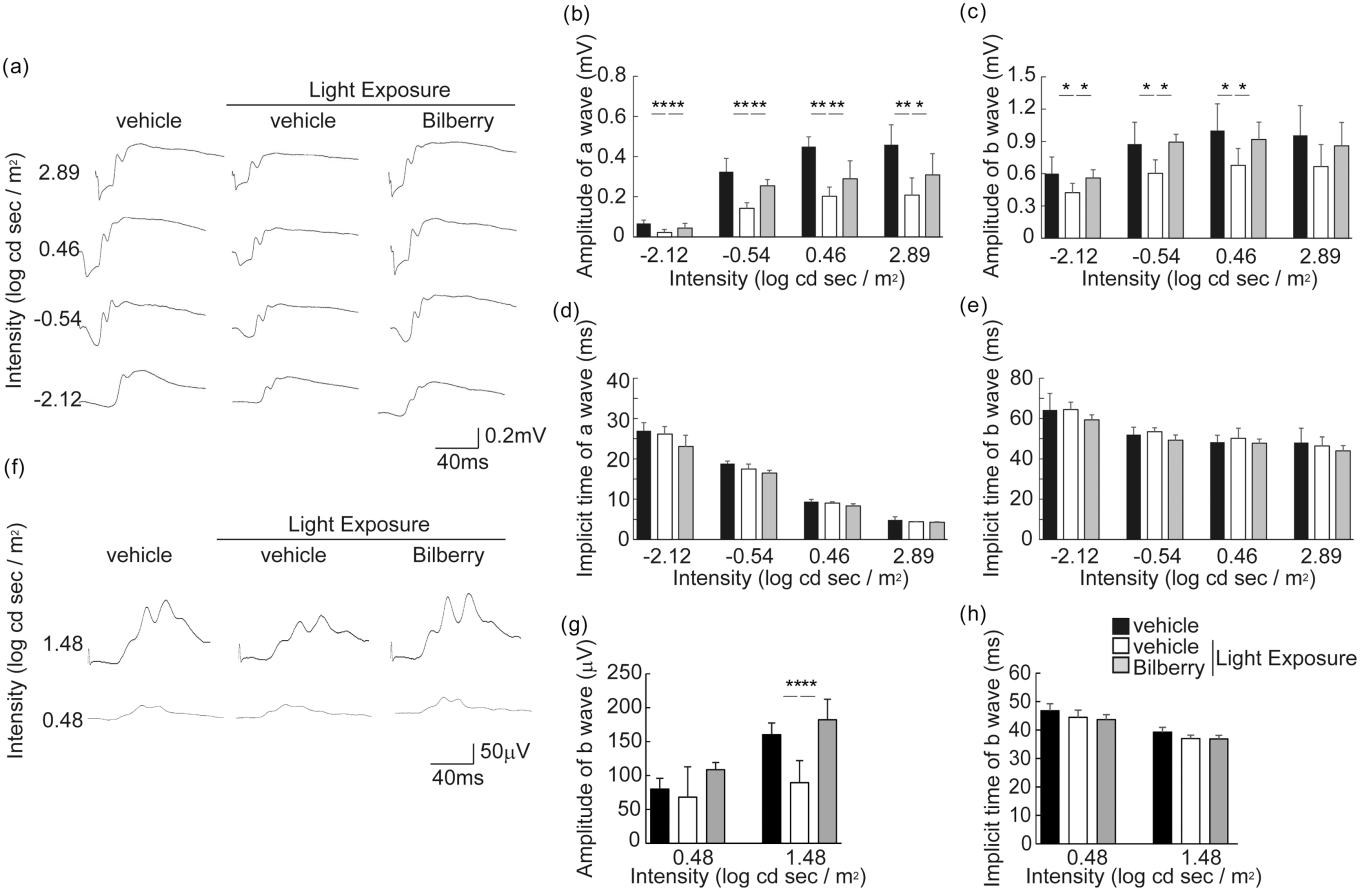
Suppression of photo-induced visual function impairment by bilberry extract. Analysis of full-field scotopic (a-e) and photopic (f-h) ERGs following light exposure. Representative waveforms of scotopic (a) and photopic (f) ERG from individual mice treated with vehicle or bilberry extract in response to one flash 4 days after light exposure. The amplitude of a- and b-waves in scotopic ERG (b and c) and b-wave in photopic ERG (g) was decreased by light exposure, and these changes were attenuated by treatment with bilberry extract. No differences were observed in a- or b-wave implicit times in both scotopic and photopic ERG (d, e and h). n = 6/ group. ERG, electroretinogram. **P < 0.01 and *P < 0.05.

The effects of bilberry extract on visual function in the light-exposed mice were determined by performing ERG 4 days after light exposure (Fig 1a–1h). In scotopic ERG (Fig 1a–1e), the amplitudes of the a- and b-waves, which show rod photoreceptor function and subsequent retinal neuronal activity, respectively, were lower in the vehicle-treated light-exposed mice than in the vehicle-treated unexposed mice kept in dim cyclic light, indicating that these illumination conditions were stressful to the retina. However, the reduction in the amplitude was attenuated by treatment with the bilberry extract (Fig 1b and 1c). In addition, the b-wave amplitude in photopic ERG, which represents cone photoreceptor system function [40, 41], decreased upon light exposure. However, this reduction was attenuated by the bilberry extract (Fig 1f and 1g). No changes were observed in the implicit times of the a- and b-waves between the groups (Fig 1d, 1e and 1h). These data suggest that the bilberry extract attenuated visual function impairment of both rod and cone photoreceptors in the photo-stressed mice.

### Reduction in photo-induced apoptotic cells by bilberry extract

**Fig 2:**
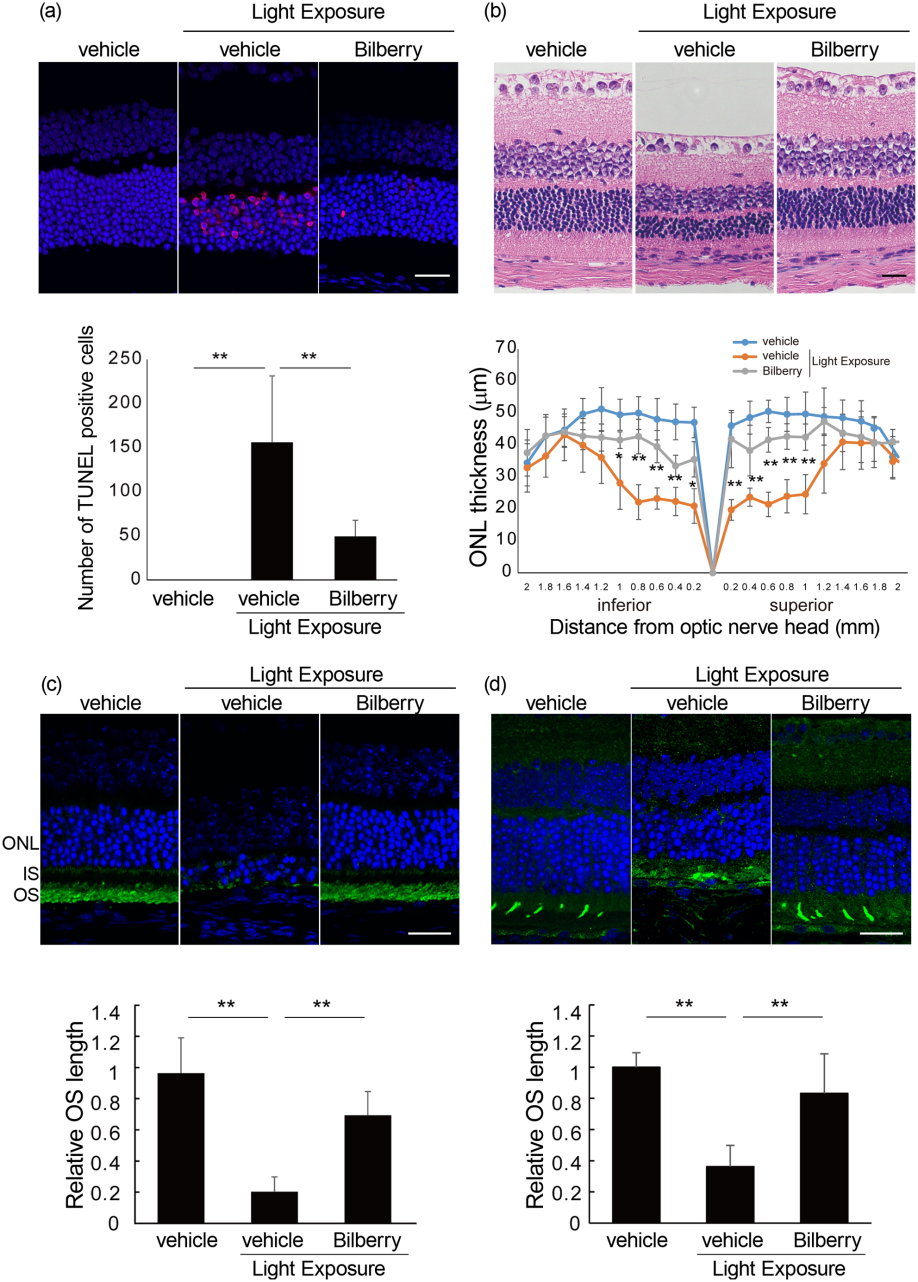
Suppression of photo-induced histological changes in the retina by bilberry extract. (a) TUNEL assay performed 2 days after light exposure. TUNEL-positive cells (red) appeared only in the ONL following light exposure. The number of apoptotic cells was significantly reduced by bilberry extract. DAPI staining of the control is shown as a guide for retinal layers. (b) H&E staining of retinal sections 4 days after light exposure. ONL thickness was lower in vehicle-treated, light-exposed mice than in vehicle-treated, non-light-exposed mice. Photo-induced ONL thinning was significantly attenuated by bilberry extract treatment. (c and d) Rhodopsin (c) and blue opsin (d) immunostaining 4 days after light exposure. The OS lengths of both rod (c) and cone (d) photoreceptors were lower in vehicle-treated light-exposed mice than in vehicle-treated non-light exposed mice. Photo-induced OS shortening was significantly attenuated by bilberry extract treatment (n = 4/ group). Scale bar, 25 μm. ONL, outer nuclear layer; DAPI, 4',6-diamidino-2-phenylindole; H&E, hematoxylin and eosin; IS, inner segment; OS, outer segment. **P < 0.01.

Next, we analyzed the effect of the bilberry extract on histological changes in the photo-stressed retina by measuring the number of TUNEL-positive and apoptotic cells 2 days after light exposure (Fig 2a). Apoptotic cells were extensively observed in the outer nuclear layer (ONL), corresponding to the photoreceptor layer of the vehicle-treated light-exposed mice, while the number was negligible in those not exposed to light. However, bilberry treatment substantially reduced the number of apoptotic cells in the photo-stressed retinas.

### Suppression of photoreceptor loss and damage by bilberry extract

The measurement of ONL thickness 4 days after light exposure revealed that it was significantly thinner in the vehicle-treated light-exposed mice than in the non-light exposed mice kept in dim cyclic light (Fig 2b). However, administration of the bilberry extract significantly attenuated ONL thinning, indicating that it attenuated the photoreceptor loss due to photo-stress. Moreover, the length of the rod outer segments (OSs), where rhodopsin was concentrated, decreased 4 days after light exposure in the vehicle-treated mice, while this phenomenon was clearly suppressed in the bilberry extract-treated mice (Fig 2c). Moreover, the length of blue cone OSs evaluated by blue opsin staining showed light-induced shortening, which was suppressed by the bilberry extract (Fig 2d). These results indicated that photo-induced photoreceptor degeneration was ameliorated by the bilberry extract.

### Antioxidative effects of bilberry extract on the retina

**Fig 3:**
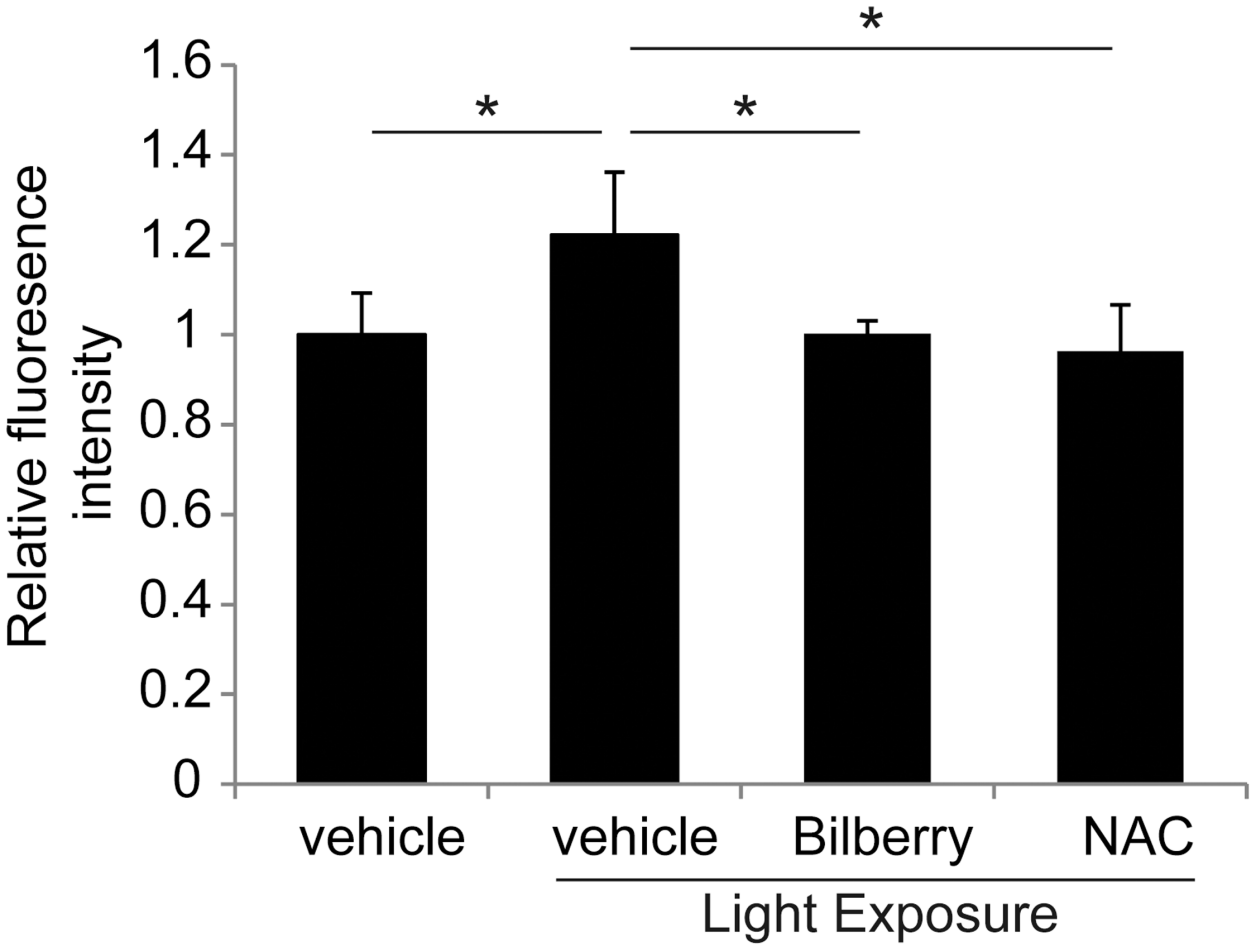
Inhibition of photo-induced ROS accumulation in the retina by bilberry extract. Retinal ROS levels were evaluated by DCFH-DA fluorescence. Photo-induced increases in retinal ROS levels were suppressed by bilberry extract and NAC treatment 6 h after light exposure; n = 4/ group. ROS, reactive oxygen species; DCFH-DA, 2’,7’-dichlorofluorescein-diacetate; NAC, N-acetyl-l-cysteine. *P < 0.05.

To evaluate the degree of retinal oxidative stress, ROS levels were measured using a DCFH-DA probe (Fig 3). The photo-induced increase in fluorescence intensity of retinas at 6 h after light exposure was significantly attenuated by treatment with the bilberry extract compared to that reported with vehicle treatment.

NAC, a well-known antioxidant that plays a role in the maintenance and metabolism of glutathione, an intrinsic antioxidant [42], has been proven to reduce ROS levels in the retina [16]. Therefore, we measured retinal ROS levels in light-exposed mice treated with NAC and showed comparable levels of reduction to that in the bilberry extract-treated animals (Fig 3). The administration protocol for NAC (via intraperitoneal injection) differed from that for bilberry extract (oral), however, ROS levels did not differ between the retinas of mice treated either with intraperitoneal or oral control PBS, both under light-exposed and non-light-exposed conditions, respectively (S1 Fig). The results suggest that the bilberry extract showed an antioxidant activity in a manner similar to that of NAC under these conditions.

### Photo-induced ER stress and the effect of bilberry extract

**Fig 4:**
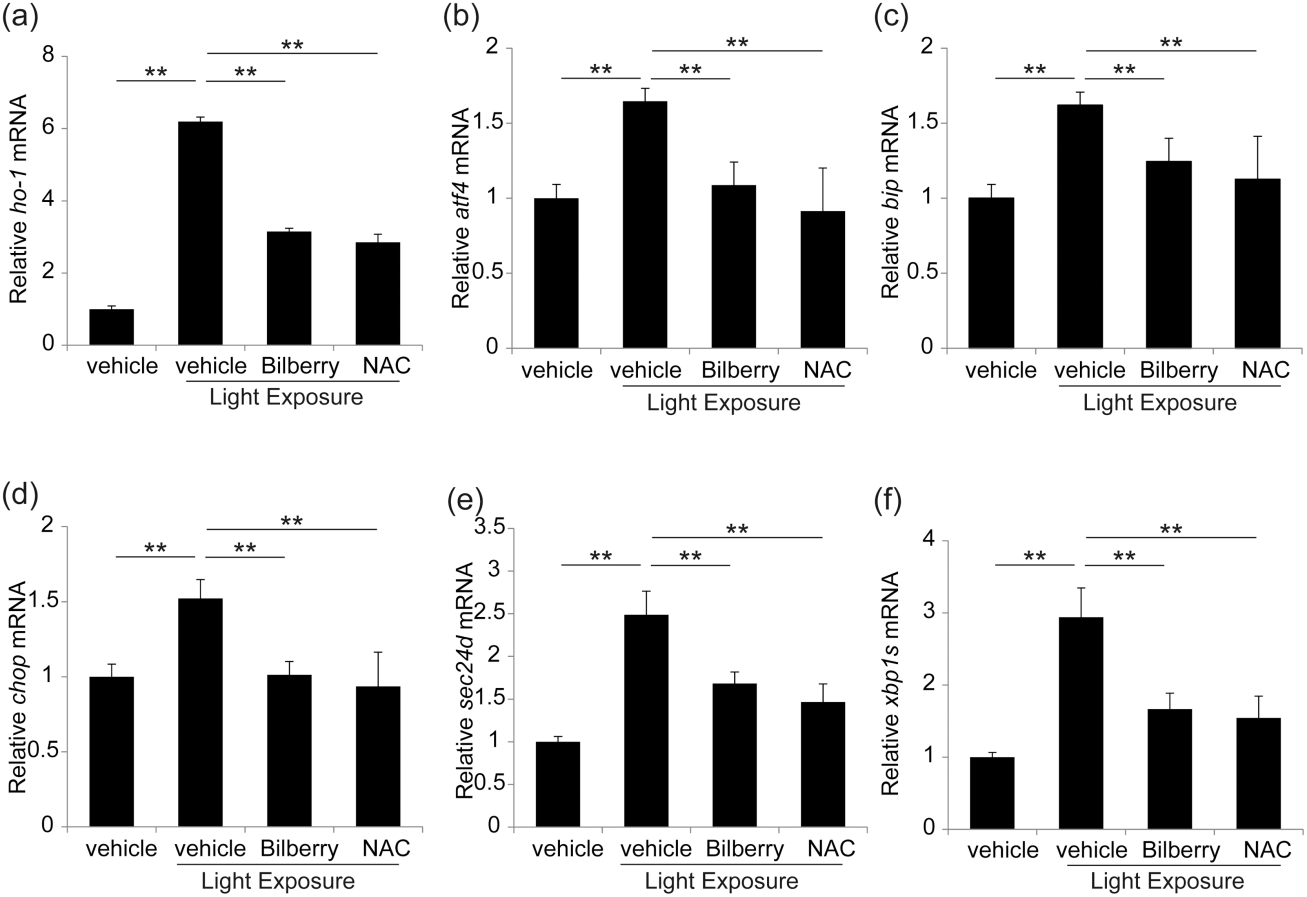
Suppression of photo-induced retinal oxidative and ER stress by both bilberry extract or NAC. (a–f) mRNA expression of markers of oxidative and ER stress was measured using real-time RT-PCR. Expression of (a) ho-1, (b) bip, (c) chop, (d) aft4, (e) xbp1s, and (f) sec24d mRNA was upregulated at 12 h in photo-stressed retinas; however, treatment with bilberry extract attenuated these increases in mRNA expression. n = 5/ group. ER, endoplasmic reticulum. **P < 0.01 and *P < 0.05.

To determine whether ER stress is involved in photo-induced retinal degeneration, we analyzed markers of ER stress using real-time RT-PCR after verifying the mRNA level of an oxidative stress marker, ho-1 [43] (Fig 4a). Interestingly, the retinal mRNA levels of bip, chop, atf4, spliced-xbp1 (xbp1s), and sec24d were all upregulated 12 h after light exposure (Fig 4b–4f). However, these increases in mRNA expression were attenuated by bilberry extract treatment, indicating that the bilberry extract attenuated ER stress in photo-stressed retinas. NAC treatment also induced a similar effect to bilberry extract treatment (Fig 4b–4f, S2 Fig).

### Suppression of photo-induced translocation of ATF4 in Müller glial cells by bilberry extract

**Fig 5:**
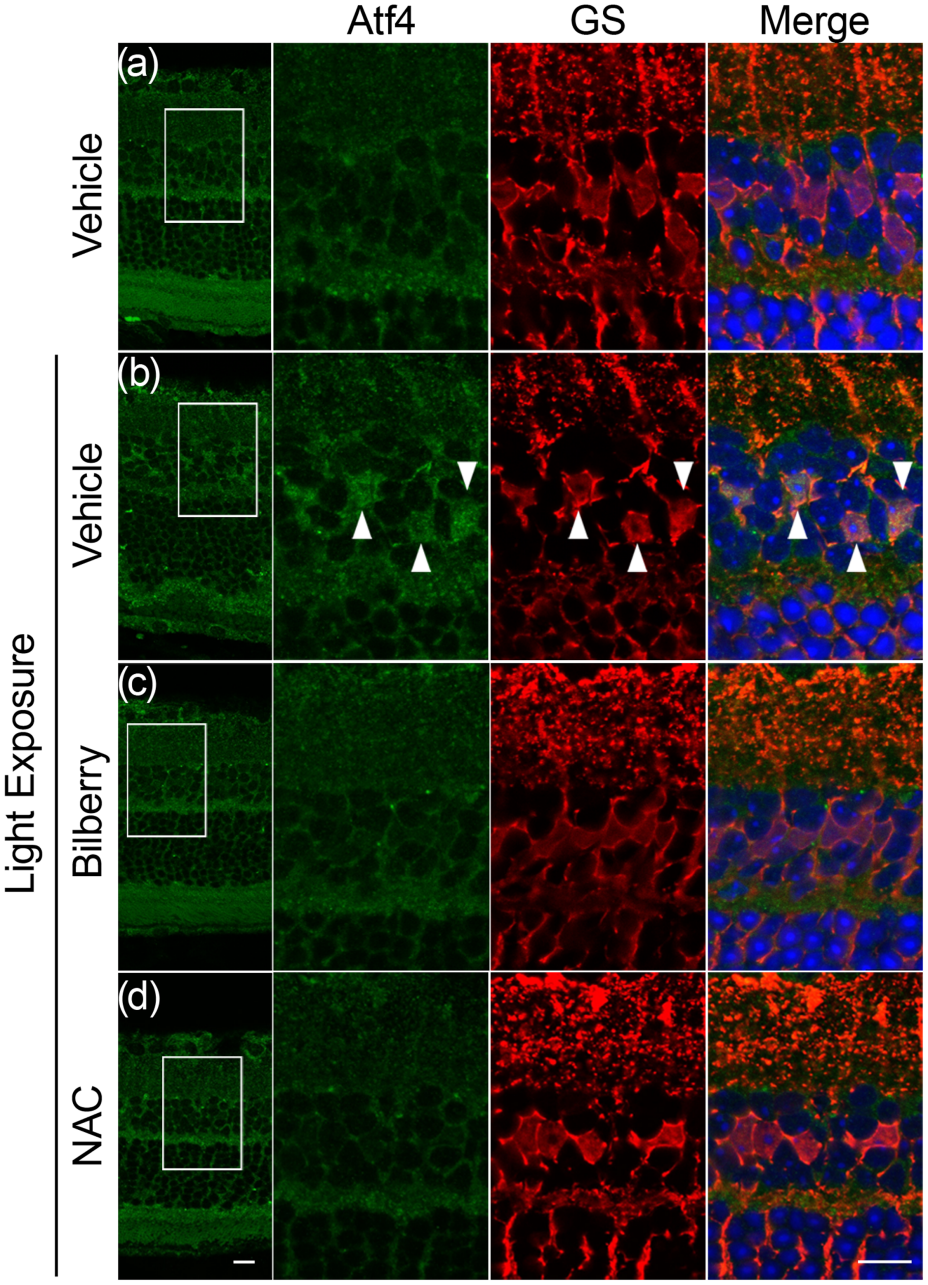
Suppression of nuclei ATF4 in Müller glial cells by bilberry extract. (a-d) Co-immunostaining for ATF4 (green) and glutamine synthetase (red), a marker for Müller glial cell, 12 h after light exposure. ATF4 staining was observed in the nuclei (DAPI, blue) of Müller glial cells after light exposure; however, this translocation was suppressed by bilberry extract (c) or NAC (d). n = 5/ group. Scale bar, 10 μm.

One pathway of ER stress induces the transcription factor ATF4 [44]. Interestingly, the translocation of ATF4 to the nuclei was observed in Müller glial cells, as determined by the expression of its marker, glutamine synthetase, 12 h after light exposure (Fig 5a and 5b). However, translocation was suppressed by both bilberry extract (Fig 5c) and NAC (Fig 5d).

### Suppression of photo-induced RPE damage by bilberry extract

**Fig 6:**
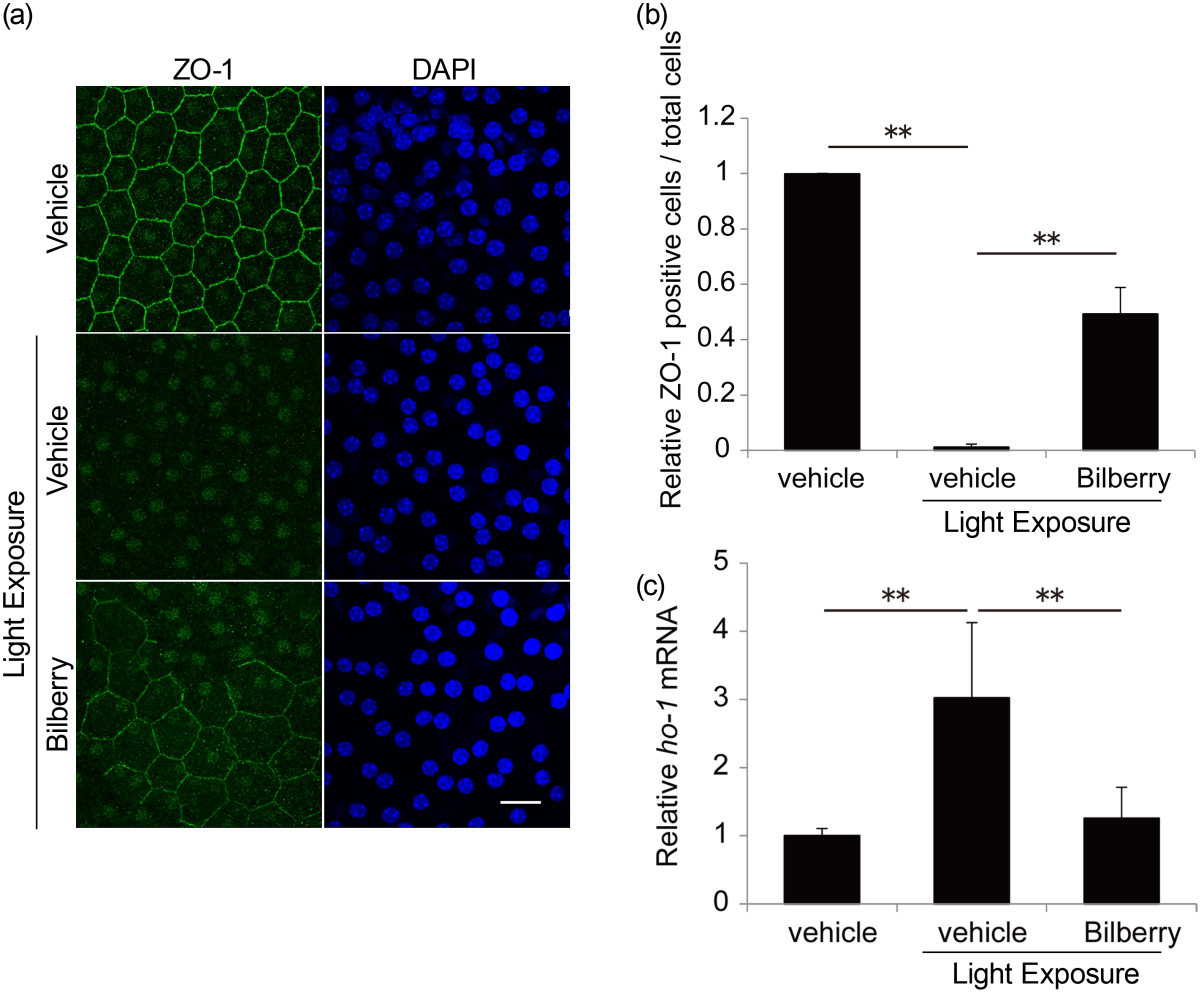
Suppression of photo-induced tight junction disruption in the RPE by bilberry extract. (a) Immunostaining for ZO-1 in flat mount samples 24 h after light exposure showed a decrease in RPE cells. (b) The number of RPE cells with an intact ZO-1 pattern at all cell edges per total RPE cells is graphically represented. (c) ho-1 mRNA expression was measured using real-time RT-PCR. RPE, retinal pigment epithelium; n = 6/ group. **p < 0.01. Scale bar, 20 μm.

Photo-induced oxidative stress disrupts the tight junctions of the RPE [45], which maintains photoreceptor conditions. Immunostaining for a tight junction marker, ZO-1, in flat mount samples 24 h after light exposure showed a decrease in RPE cells with an intact ZO-1 pattern at all cell edges per total RPE cell (Fig 6a and 6b). During the study time-period, there was no obvious nuclear condensation in the RPE cells of animals exposed to light and treated with either vehicle or bilberry extract, suggesting a lack of RPE cell death. Under this condition, the expression of an oxidative stress marker, ho-1, was increased by light exposure but suppressed by bilberry extract (Fig 6c). There was no difference in the mRNA levels of ER stress markers under this condition (data not shown).

## Discussion

In this study, we showed the suppression of light-induced visual impairment (Fig 1) and rod and cone photoreceptor damage/apoptosis (Fig 2) by the administration of bilberry extract, which reduced light-induced retinal ROS levels and ER stress (Figs 3 and 4, respectively). A standard antioxidant, NAC, also similarly attenuated ER stress caused by light exposure (Figs 3 and 4, respectively). One ER stress marker, ATF4, showed a photo-induced nuclear translocation in Müller glial cells, which was suppressed by both bilberry extract and NAC (Fig 5). The photo-induced disruption of tight junctions in the RPE was also attenuated by bilberry extract (Fig 6).

The scotopic ERG results showed that both the a- and b-wave amplitudes decreased after light exposure, indicating that rod photoreceptor function was impaired. The a-wave abnormalities represented an original change in the photoreceptors, and b-wave abnormalities most likely were responsive to the decrease in the input from the photoreceptors to downstream retinal neurons, thus reflecting the visual response to damaged photoreceptors. Moreover, the photopic ERG recorded after light adaption showed impairment of cone photoreceptor function. In photopic ERG, the b-wave is commonly regarded as an indicator of cone system function because the a-wave amplitude is too small to evaluate, and because only cone photoreceptors (and not rod photoreceptors) respond under photopic conditions [40, 41]. This photoreceptor disorder was further evidenced by the TUNEL-positive cells in the photoreceptor layer preceding visual function impairment and a clear decrease in ONL thickness representing photoreceptor loss. The shortening of both rod and cone OSs demonstrated a reduction in photon receptors in the remaining photoreceptors, and was likely involved in the impaired photoreceptor responses observed in the ERG. These results showing photo-induced rod photoreceptor degeneration, including visual function impairment and histological changes, were consistent with the results of previous studies [13, 15, 16, 33, 34]. Similar results were obtained in the current study with respect to cone photoreceptors.

Bilberry extract treatment attenuated all the observed photo-induced photoreceptor degeneration, including the increased ROS levels in the retina of photo-stressed mice. Furthermore, the ROS levels in the bilberry extract-treated retinas were almost equal to those observed in the retinas of light-exposed mice treated with a dosage of NAC previously reported to suppress photo-induced photoreceptor degeneration [16]. These data and previous reports showing *in vitro* antioxidant effects support the idea that the neuroprotective effects of the bilberry extract against photoreceptor apoptosis and degeneration observed in the current study may be attributable to its antioxidant properties.

Increases in retinal ROS were observed as early as 6 h after light exposure, which was suppressed by the bilberry extract. The oral consumption of bilberry extract rapidly increased the serum levels of the major component of bilberry extract, anthocyanins [46], which peaked 15–30 min after ingestion in mice [47]. The rapid systemic absorption of anthocyanins may have contributed to the effective suppression of the early increase in retinal ROS levels after light exposure, and therefore attenuated photoreceptor degeneration.

Although the increase in retinal ROS levels were as much as 20%, this amount may have been sufficient to cause retinal degeneration. The retinal protective effects of bilberry extract and NAC were most likely attributable to the removal of locally accumulated ROS. Alternatively, Narimatsu *et al*. showed that angiotensin II type 1 receptor (AT1R) signaling occurs upstream of photo-induced ROS generation in the retina [16]. Furthermore, AT1R blockade efficiently suppressed light-induced photoreceptor apoptosis, OS shortening, and functional impairment [16]. A subgroup of the anthocyanins, proanthocyanidins, has been reported to bind to and inhibit AT1R [48, 49]. It would be interesting to study the effects of the bilberry extract on AT1R and ROS generation in future research.

Treatment with the bilberry extract, as well as NAC, reduced ROS accumulation in the retina and attenuated ER stress, suggesting that photo-induced ER stress was activated by ROS. This was consistent with a previous report on brain neuronal death showing that ER stress in an ischemia/reperfusion model was suppressed in transgenic mice overexpressing superoxide dismutase 1, an antioxidant enzyme [50]. The relationship between oxidative and ER stress may be explained by the oxidation of both ER-resident and other proteins transported by the ER, which may activate the UPR [51, 52], although the involvement of a ROS-independent pathway in ER stress activation cannot be excluded after light exposure. Therefore, reducing oxidative stress may be important for maintaining normal ER functions, including UPR.

Retinitis pigmentosa is a photoreceptor degenerative disease caused by genetic mutations. In a mouse model of *rd10* retinitis pigmentosa, the suppression of ER stress by inhibiting adenosine triphosphatase (ATPase) activity using a valosin-containing protein attenuated photoreceptor loss [53]. Moreover, photoreceptors with a gene mutation for rhodopsin differentiated from induced pluripotent stem (iPS) cells derived from an affected patient showed an increase in ER stress and apoptosis, which were attenuated by rapamycin [54], suppressing ER stress [55], suggesting the involvement of ER stress in retinitis pigmentosa-induced photoreceptor death. In the current study, light exposure induced CHOP in the retina, suggesting that the misfolded and unfolded proteins may have increased in the retina regardless of the presence of mutation. One of the mechanisms underlying the progression of retinitis pigmentosa caused by light exposure may involve a scenario where photo-induced ER stress may easily exceed the capacity of UPR in situations where UPR is already in effect owing to mutation-coded abnormal proteins.

In addition, Müller glial cells were involved in photo-induced ER stress in the current study, suggesting the involvement of impaired photoreceptor-Müller glia interaction which induced abnormality in photoreceptor maintenance.

The disruption of tight junctions in the RPE by photo-induced oxidative stress was also suppressed by bilberry extract, although the pathway may not involve ER stress (data not shown). Because RPE also maintains photoreceptor survival [56], bilberry extract may also have exerted a protective effect on photoreceptors by protecting RPE conditions.

In this study, the reduction of oxidative stress subsequently reduced ER stress and apoptosis in photo-stressed retinas. Moreover, the micronutrient-containing bilberry extract supplement clearly acted as an antioxidant in the treatment of retinal oxidative stress, reducing ER stress and apoptotic signaling (S3 Fig). The current study will facilitate the elucidation of photo-stress pathogenesis and development of a therapeutic approach for preventing the progression of retinal degeneration.

## Supporting information

S1 Fig Equivalent ROS levels after treatment with control vehicle by intraperitoneal or oral administration. Retinal ROS levels evaluated by DCFH-DA fluorescence were similar following treatment with control vehicle by either the intraperitoneal (IP) or oral route, 6 h after light exposure; n = 4/ group. ROS, reactive oxygen species; DCFH-DA, 2’,7’-dichlorofluorescein-diacetate. *P < 0.05.
(TIF)

S2 Fig Equivalent levels of oxidative and ER stress markers after treatment with control vehicle by intraperitoneal or oral administration. (a–f) mRNA expression of markers of oxidative and ER stress was measured using real-time RT-PCR. Expression of (a) ho-1, (b) bip, (c) chop, (d) aft4, (e) xbp1s, and (f) sec24d mRNA was similar under the control conditions of oral or intraperitoneal (IP) PBS administration for 12 h in photo-stressed retinas. n = 5/ group. ER, endoplasmic reticulum. **P < 0.01 and *P < 0.05.
(TIF)

S3 Fig Model of the mechanisms of photo-induced visual function impairment and the protective role of bilberry extract. Excessive light exposure causes ROS accumulation, which induces ER stress to initiate photoreceptor apoptosis and degeneration and subsequent visual function impairment. A pathway for ROS-independent ER stress could not be excluded. ROS-dependent RPE changes may also cause photoreceptor disorder. Bilberry extract at least partly reduced ROS and ER stress to protect photoreceptors and visual function.
(TIF)
