# Supplementary figures and images for: Neuroprotective effect of bilberry extract in a murine model of photo-stressed retina
Source: PLoS One. 2017 Jun 1;12(6):e0178627. doi: 10.1371/journal.pone.0178627 (PMC5453571; doi:10.1371/journal.pone.0178627)

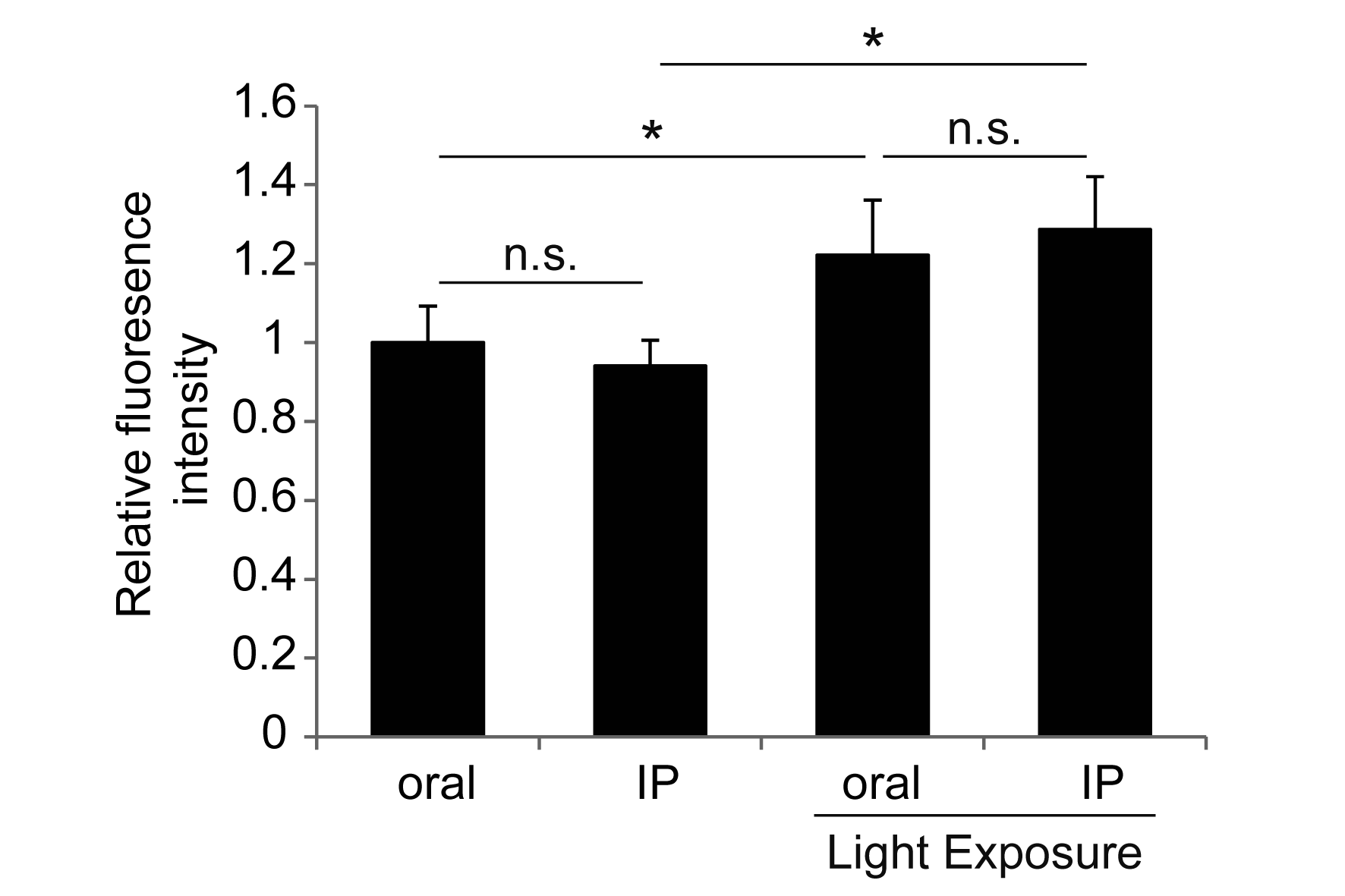

Supplement: S1 Fig — Retinal ROS levels evaluated by DCFH-DA fluorescence were similar following treatment with control vehicle by either the intraperitoneal (IP) or oral route, 6 h after light exposure; n = 4/ group. ROS, reactive oxygen species; DCFH-DA, 2’,7’-dichlorofluorescein-diacetate. *P < 0.05. (TIF) [file pone.0178627.s001.tif]

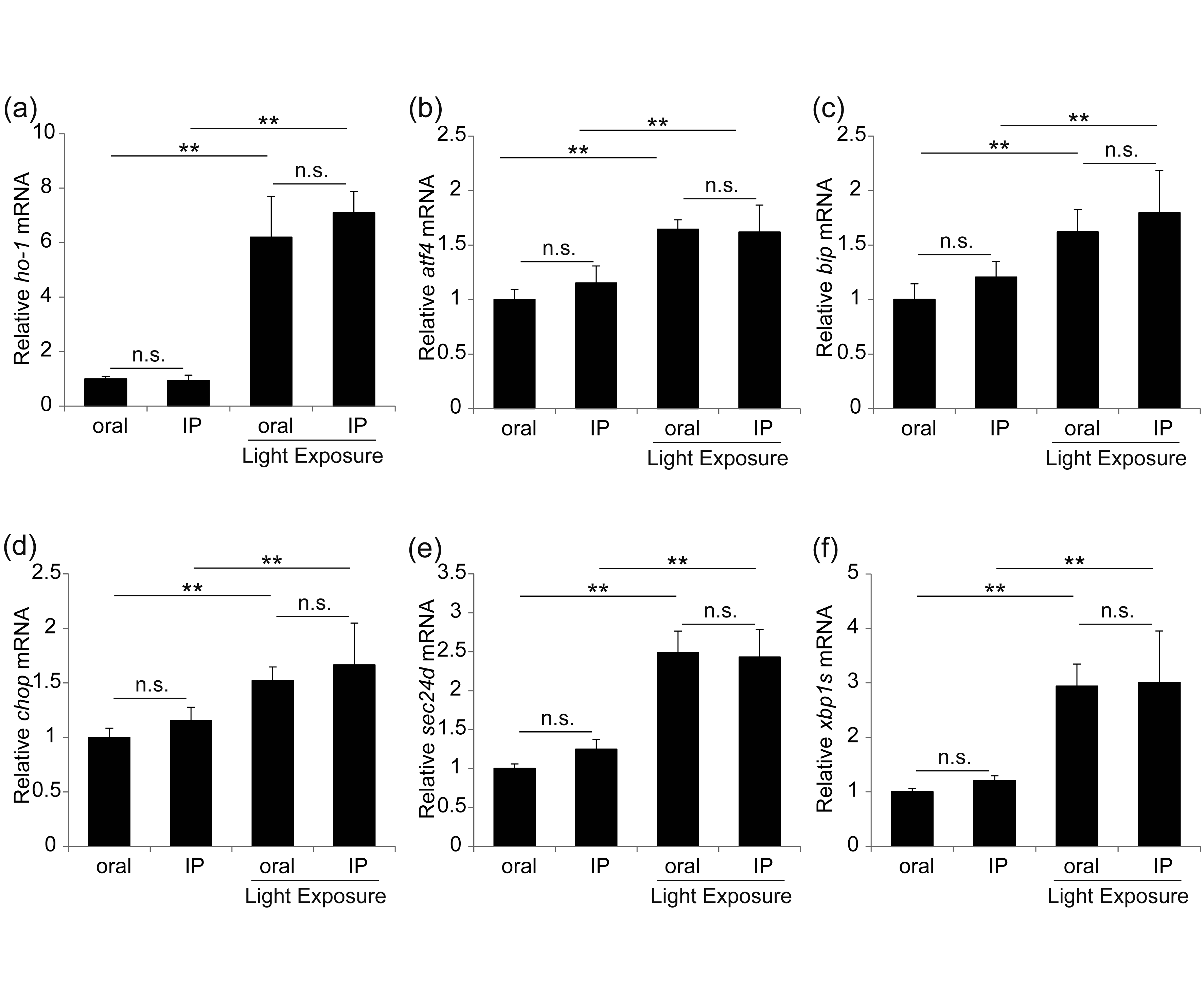

Supplement: S2 Fig — (a–f) mRNA expression of markers of oxidative and ER stress was measured using real-time RT-PCR. Expression of (a) ho-1, (b) bip, (c) chop, (d) aft4, (e) xbp1s, and (f) sec24d mRNA was similar under the control conditions of oral or intraperitoneal (IP) PBS administration for 12 h in photo-stressed retinas. n = 5/ group. ER, endoplasmic reticulum. **P < 0.01 and *P < 0.05. (TIF) [file pone.0178627.s002.tif]

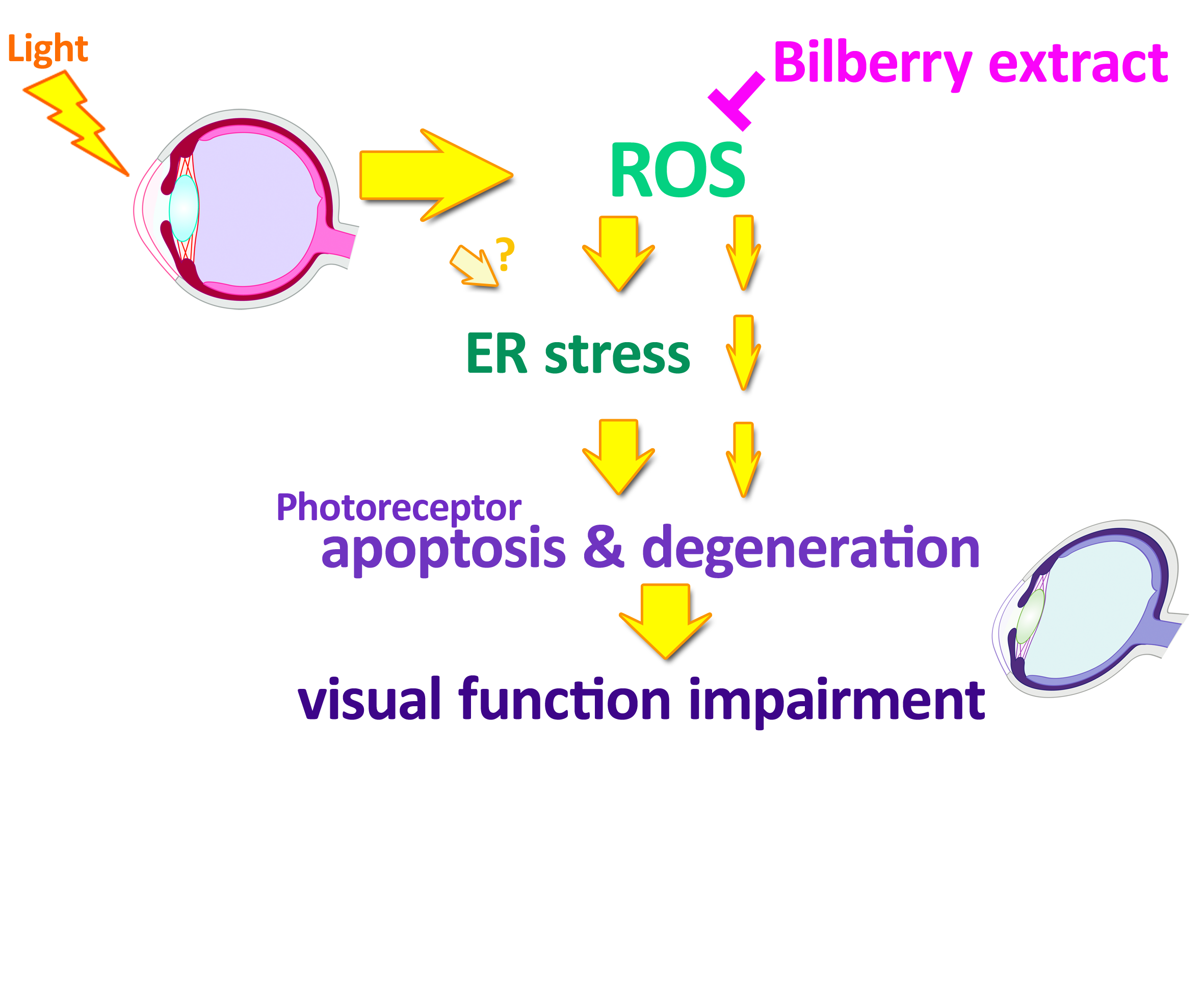

Supplement: S3 Fig — Excessive light exposure causes ROS accumulation, which induces ER stress to initiate photoreceptor apoptosis and degeneration and subsequent visual function impairment. A pathway for ROS-independent ER stress could not be excluded. ROS-dependent RPE changes may also cause photoreceptor disorder. Bilberry extract at least partly reduced ROS and ER stress to protect photoreceptors and visual function. (TIF) [file pone.0178627.s003.tif]
